# Initial Experience of CT-Based Online Adaptive Radiotherapy for Nasopharyngeal Carcinoma With a Novel Integrated Platform: A Case Report

**DOI:** 10.7759/cureus.69262

**Published:** 2024-09-12

**Authors:** Yu-Xian Yang, Lin Li, Gangyu Wang, Xiaobo Jiang, Hua Li, Le-cheng Jia, Guanqun Zhou, Ying Sun

**Affiliations:** 1 Department of Radiation Oncology, Sun Yat-sen University Cancer Center, Guangzhou, CHN; 2 Real-time Laboratory, Shenzhen United Imaging Research Institute of Innovative Medical Equipment, Shenzhen, CHN

**Keywords:** ct-guided online adaptive radiotherapy, dosimetric benefit, feasibility, nasopharyngeal carcinoma, novel integrated platform

## Abstract

Intensity-modulated radiation therapy (IMRT) improves tumor control and reduces long-term radiation-induced complications of patients with nasopharyngeal carcinoma (NPC), contingent upon accurate contouring and precise delivery of treatment plans. Online adaptive radiotherapy (ART) involves real-time treatment plan modification based on the variations in targets and organs at risk (OARs) to uphold treatment planning accuracy. This study describes the first reported case of fan beam computed tomography (FBCT)-guided online ART for NPC using a novel integrated platform. Online ART was performed at the 25th fraction in this case, as tumors and the patient’s anatomy were observed to regress inter-fractionally, necessitating adjustments to the contours based on the anatomy of the day. Online ART plan optimized target volume coverage while reducing doses to OARs. Notably, online ART significantly improved radiotherapy efficiency. This patient achieved a clinical complete response 12 weeks post-treatment, with Epstein-Barr virus DNA levels reduced to 0 copies/ml. Currently, the patient is alive without evidence of high-grade toxicity or local recurrence at approximately 10 months post-treatment. This case confirms the feasibility and dosimetric benefit of online ART for NPC using a novel integrated platform. Further research is needed to confirm its clinical benefits.

## Introduction

Nasopharyngeal carcinoma (NPC) is prevalent in certain regions, particularly in East and Southeast Asia [1,2]. Intensity-modulated radiation therapy (IMRT) provides excellent locoregional control and spares of organs at risk (OARs) in patients with NPC [3]. A radiotherapy treatment plan is typically established before initiating the radiotherapy and remains static throughout the 6-8 weeks treatment course. However, patients and tumors undergo continuous changes during this period [4-6]. Steep dose gradients of IMRT make it vulnerable to anatomical variations and setup errors, potentially leading to suboptimal tumor dose delivery or excessive irradiation of OARs [7-10].

Adaptive radiotherapy (ART) is the process of modifying a treatment plan based on the variations of targets and OARs to maintain the initial intent of the radiation oncologist [11]. Online ART involves reimaging, recontouring, and replanning based on the patient’s anatomy of the day, all while the patient is on the treatment table, effectively addressing anatomical variations and setup errors [12]. The development of artificial intelligence (AI) technology for fast and accurate contouring and planning, coupled with the integration of computed tomography (CT) with linear accelerators, has facilitated the implementation of cone beam computed tomography-guided online ART [13-18]. Although no clinical trials have established the superiority of online ART over mid-course replanning, online ART offers the advantage of rapid adjustments within minutes. This capability helps minimize the impact of anatomical changes on treatment accuracy and allows for multiple quick adjustments, potentially enhancing the overall precision of radiotherapy.

The complexity of NPC radiation planning presents challenges for the application of online ART. The specific benefits of online ART for NPC patients are not yet fully understood. This study explores a unique case, reporting on a patient who was successfully treated with fan beam computed tomography (FBCT)-guided online ART using a novel integrated platform, with a particular focus on the feasibility and dosimetric benefit of this approach.

## Case presentation

Baseline patient information

**Figure 1:**
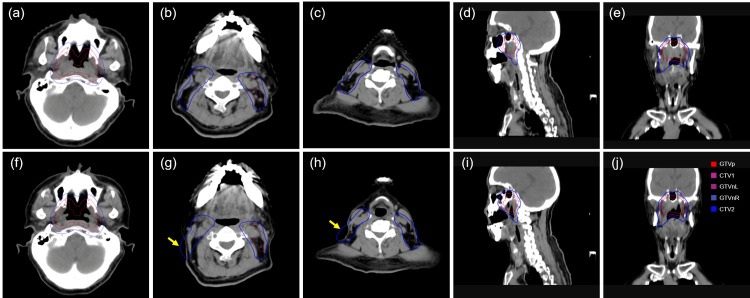
CT images show contour of target volumes in axial, sagittal, and coronal views (a-e) CT images acquired before radiotherapy show pre-radiotherapy target volume contours. (f-j) CT images acquired during the online ART fraction show contours rigidly registered from the pre-radiotherapy CT images. Target contours with GTVp in red, GTVn_L in purple, GTVn_R in light blue, CTV1 in scarlet, CTV2 in blue. CT: Computed tomography; ART: Adaptive radiotherapy; GTVp: Gross tumor volume at the primary site; GTVn_L: Left involved cervical lymph node; GTVn_R: Right involved cervical lymph node; CTV1: High-risk clinical tumor volume; CTV2: Low-risk clinical tumor volume

A 74-year-old male patient first presented to radiation oncology due to epistaxis and nasal congestion for half of a year, with a World Health Organization performance status of 0. Nasopharyngeal fiberoptic endoscopy showed tumors in both bilateral pharyngeal recesses and the posterior roof of the nasopharynx, and a biopsy confirmed undifferentiated non-keratinizing carcinoma of the nasopharynx. Epstein-Barr virus (EBV) DNA was 268 copies/ml. Magnetic resonance imaging (MRI) showed invasion of bilateral pharyngeal recesses, posterior nasal passages, bilateral levator veli palatini muscles, and tensor veli palatini muscles, and suspected invasion of the base of the sphenoid bone. No evidence showed lymph node involvement or distant metastases. This patient was staged as T3N0M0 III according to the 8th Union for International Cancer Control and the American Joint Committee on Cancer (Figure 1a-1e). After discussion with the multidisciplinary team, the patient was recommended to receive radiotherapy with weekly nimotuzumab.

Treatment planning and delivery

The patient was positioned supine with the head in a neutral position and was immobilized using customized thermoplastic head, neck, and shoulder devices, along with individual oral stents. MRI simulation was performed using a 75 cm big-bore MRI simulator (uMRI Omega, UIH, Shanghai, China). MRI sequences included unenhanced, contrast-enhanced, fat-suppressed T1-weighted sequences, and unenhanced T2-weighted sequences. MRI images were acquired with a 3 mm layer thickness, covering the area from the superior border of the frontal sinus to 2 cm below the clavicle. A diagnostic quality FBCT scan was obtained on a novel integrated linear accelerator nameduRT-linac 506c (Shanghai United Imaging Healthcare Co., Ltd., Shanghai, China) with the same range and layer thickness as MRI simulation. The uRT-linac 506c platform integrates a C-arm linear accelerator with a diagnostic-quality 16-slice CT imager. This integration enables a seamless workflow from simulation to treatment, greatly enhancing efficiency. Key features include auto-segmentation and auto-planning based on daily FBCT images, enabling online ART. Additionally, it supports *in vivo* verification with an electronic portal imaging device, allowing for real-time gamma passing rate calculations during treatment. These innovations collectively offer a comprehensive solution for advanced radiotherapy, setting this platform apart from conventional systems [19].

Delineation was performed by the integrated treatment planning and oncology information system (Shanghai United Imaging Healthcare Co., Ltd., Shanghai, China). Target volumes, including the gross tumor volume at the primary site (GTVp), involved cervical lymph nodes (GTVn), high-risk clinical target volume (CTV1), and low-risk clinical target volume (CTV2), were delineated according to consensus guidelines [20,21]. A uniform 3 mm expansion was applied to the gross tumor volumes (GTVs) and clinical target volumes (CTVs) to create the planning target volumes (PTVs).

The prescribed doses of this patient were 70 Gy (2.12 Gy/fraction) to the PTV of GTVp, 66 Gy (2.00 Gy/fraction) to the PTV of GTVn, 60 Gy (1.82 Gy/fraction) to the PTV of CTV1, and 54 Gy (1.64 Gy/fraction) to the PTV of CTV2. These doses were delivered using the simultaneous integrated boost technique, with all PTVs irradiated simultaneously for 33 daily fractions, five fractions per week. We used volumetric modulated arc therapy (VMAT) with two full arcs for all patients with NPC. The dose calculation algorithm is based on the Monte Carlo algorithm. The clinical goals for optimizing the initial plan and the online ART plan are outlined in Table 1. The uRT-linac 506c is also used to provide low-dose FBCT for image-guided radiation therapy (IGRT) and perform treatment delivery.

**Table 1:** Clinical goals used for planning GTV: Gross tumor volume; PTV: Planning target volume & - Dose received by 100% of the target volume * - Maximum point dose to the volume § - Percent of volume receiving ≥ 60 Gy ‡ - Dose received by 0.03 cm^3^ of the volume † - Mean dose to the volume ♯ - Dose received by 2 cm^3^ of the volume @ - Percent of volume receiving ≥ 30 Gy

Structure	Goal	Priority
GTV	D100% § > 98% Rx	2
PTV	V100% * ≥ 97.5%	2
	Dmax # < 107% Rx	
Brainstem	Dmax ≤ 7000 cGy	1
	V60 Gy $ ≤ 10%	
SpinalCord	Dmax ≤ 4500 cGy	1
Eye_L(R)	D0.03 cc ‡ ≤ 5400 cGy	2
	Dmean † ≤ 3500 cGy	
Lens_L(R)	D0.03 cc ≤ 600 cGy	2
Chiasm	D0.03 cc ≤ 5400 cGy	2
TemporalLobe_L(R)	D0.03 cc ≤ 7500 cGy	2
OpticNerve_L(R)	Dmax ≤ 7000 cGy	2
Thyroid	Dmean ≤ 3000 cGy	2
Mandible_L(R)	D2cc ♯ ≤ 7000 cGy	3
OralCavity	Dmean ≤ 4000 cGy	3
Parotid_L（R）	V30 Gy @ ≤ 50%	3
	Dmean ≤ 2600 cGy	
Submandibular_L(R)	Dmean ≤ 3500 cGy	3

Online adaptive planning

The patient was positioned on the couch and was placed with lead markers for alignment. The new FBCT scanning range and layer thickness were consistent with the initial treatment. After registering new FBCT sequences with planning FBCT sequences, the GTVp on the planning FBCT was then rigidly copied onto the new FBCT sequences. GTVn, CTV1, CTV2, and 35 OARs were contoured on the new FBCT sequences. A modified 3D-UNet was applied for CTV1, CTV2, GTVn, and OARs auto-segmentation. To guide the auto-segmentation of GTVn and CTVs, we introduced the GTVp (input constraints) and OARs (output constraints) as empirical guides. This innovative approach considers the relationships between target volumes and surrounding OARs and interactions between different target volumes, resulting in contours that better align with clinical requirements. For OARs, a multi-mask auto-segmentation network was introduced to improve the speed of the segmentation algorithm. The rigidly copied GTVp and AI-generated targets and OARs were reviewed and modified by radiation oncologists based on the anatomy of the day.

Given the challenges of planning design for NPC due to the tumor’s irregular shape and proximity to critical structures, previously treated high-quality plans were used as training data to develop a deep-learning model for predicting 3D dose distribution. The plan optimization algorithm utilized the predicted 3D dose distribution and a clinical goal sheet as inputs. To directly generate a deliverable and clinically acceptable plan without user intervention, the algorithm automatically integrated various optimization strategies and applied them as necessary. The algorithm includes a predictive adjustment feature that supports the manual adjustment of the AI-generated plan, providing greater flexibility and convenience. The medical physicist and radiation oncologist approved and evaluated the AI-generated plan. The initial and adaptive plans were assessed using the patient’s anatomy of the day (IGRT and ART plans) and compared using dose-volume histograms.

Dosimetric and treatment data

**Figure 2:**
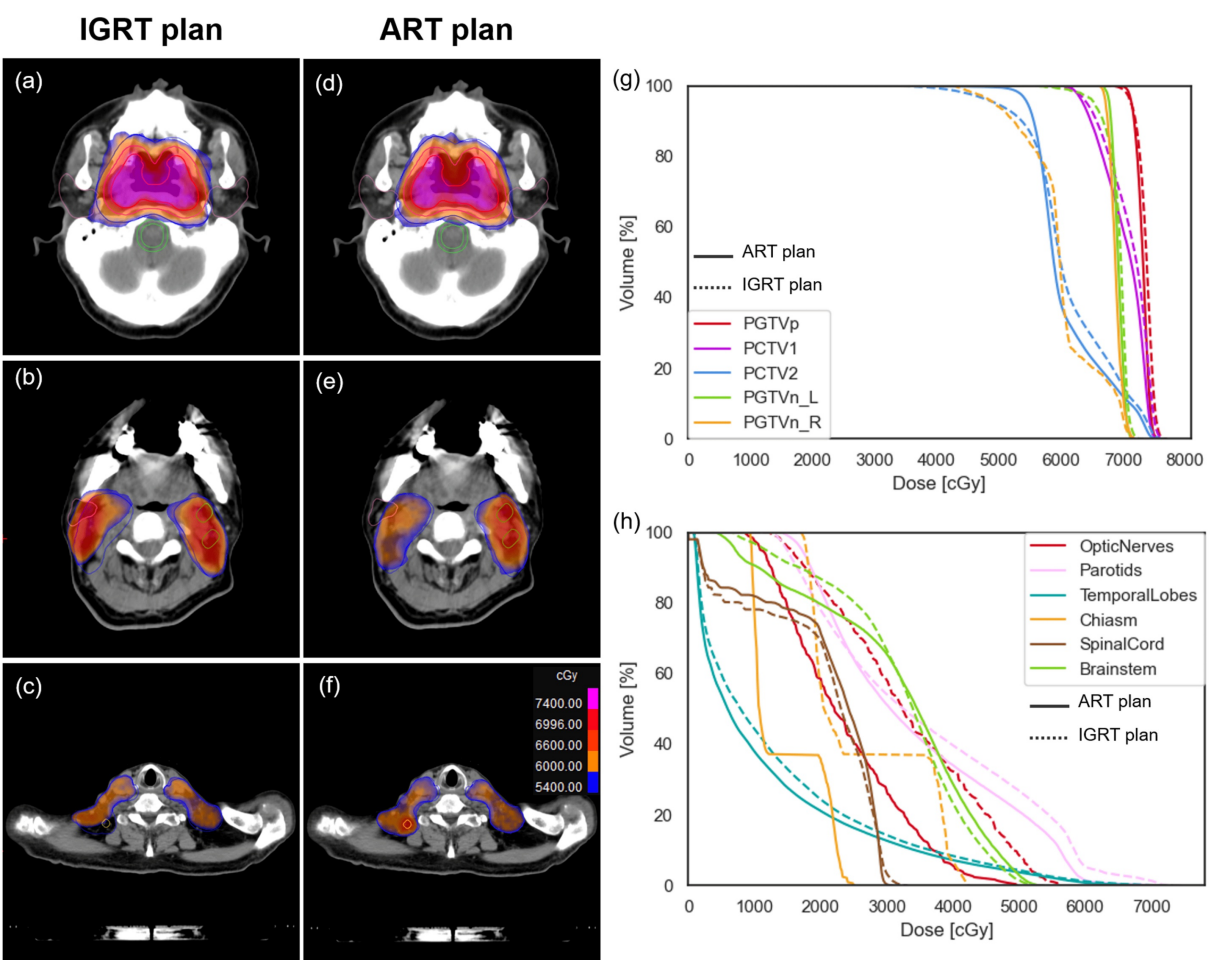
Dose distribution comparison between (a-c) IGRT and (d-f) ART plans The cumulative DVH plot of target volumes and OARs are shown in (g) and (h), respectively. The solid line represents the ART plan and the dotted line represents the IGRT plan. IGRT: Image-guided radiation therapy; ART: Adaptive radiotherapy; DVH: Dose-volume histogram; OAR: Organs at risk

After being treated with 24 fractions of radiotherapy, this patient experienced a 10% weight loss, a 9% reduction in the horizontal diameter of the nasopharyngeal and neck regions, and a 23% regression of the primary tumor, resulting in a significant mismatch between the target volumes and anatomy (Figure 1f-1j). Consequently, the radiation oncologist decided to proceed with online ART at the 25th fraction. Figure 2 and Table 2 show the comparison of dose distribution between the IGRT and the ART plans. The percentage of target volume receiving ≥ 100% of the prescribed dose for PGTVp_6996 cGy, PGTVn_6600 cGy, PCTV1_6006 cGy, and PCTV2_5412 cGy were 98.9%, 75%, 99.7%, and 90% in the IGRT plan, and 99.8%, 100%, 100%, and 97.5% in the ART plan, respectively. Compared to the IGRT plan, the doses to most OARs, such as the spinal cord, temporal lobes, chiasm, optic nerves, eyes, lens, cochleae, mandibles, temporomandibular joints, oral cavity, Eustachian tube bones, internal auditory canals, larynx_supraglottic, larynx_glottic and vestibulsemis were lower in the ART plan.

**Table 2:** Comparison of dosimetric parameters between IGRT and adaptive plans for a specific fraction IGRT: Image-guided radiation therapy; ART: Adaptive radiotherapy; GTVp: Gross tumor volume at the primary site; GTVn: Involved cervical lymph nodes; PGTVp: Planning target volume of the gross tumor at the primary site and the involved retropharyngeal lymph node; PGTVn: Planning target volume of the involved cervical lymph node; PCTV1: Planning target volume of the high-risk clinical target volume; PCTV2: Planning target volume of the low-risk clinical target volume; TM: Temporomandibular; ET: Eustachian; IACs: Internal auditory canals § - Dose received by 100% of the target volume * - Percent of volume receiving ≥ 100% of the prescribed dose ‡ - Dose received by 98% of the target volume † - Percent of volume receiving ≥ 98% of the prescribed dose ♯ - Percent of volume receiving ≥ 95% of the prescribed dose @ - Dose received by 0.03cm3 of the volume & - Mean dose to the volume ^ - Maximum point dose to the volume # - Dose received by 2% of the volume

Structures	Clinical Goal	IGRT	ART
GTVp	D100% § > 6856.1 cGy	7058.1	7014.4
GTVn	D100% > 6468 cGy	4922.7	6813.5
PGTVp	V100% * ≥ 97.5%	98.9	99.8
	D100% ≥ 6646.2 cGy	6707.7	6870.5
	D98% ‡ ≥ 6996 cGy	7047.4	7091
PGTVn	V100% (%) ≥ 97.5%	75	100
	V98% † (%) ≥ 98%	78	100
	V95% ♯ (%) ≥ 100%	80	100
PCTV1	V100% (%) ≥ 97.5%	99.7	100
	D100% ≥ 5705.7 cGy	5581.4	5936.2
	D98% ≥ 6006 cGy	6261.4	6249.5
PCTV2	V100% ≥ 97.5%	90	97.5
	D100% ≥ 5130 cGy	3244.2	4805.5
	D98% ≥ 5400 cGy	4496.6	5388.3
SpinalCord	D0.03cc @ ≤ 5000 cGy	3230.4	3026.9
BrainStem	D0.03cc ≤ 6000 cGy	5258.5	5278.6
TemporalLobes	D0.03cc ≤ 7200 cGy	7211.5	7039
Chiasm	D0.03cc ≤ 6000 cGy	4062.1	2302.8
OpticNerves	D0.03cc ≤ 6000 cGy	5157.4	4026.2
Eyes	Dmean & < 3500 cGy	1319.5	864.2
Lens	D0.03cc ≤ 800 cGy	1107.6	676.7
Cochleae	Dmean < 5500 cGy	5379.6	5165.5
Parotids	Dmean < 3000 cGy	3437.3	3586.6
Submandibulars	Dmean < 3500 cGy	4799.5	4859.8
Pituitary	Dmax ^ < 6000 cGy	5951.5	5964
Thyroid	Dmean < 4500 cGy	4597.2	4621.7
Mandibles	D0.03cc < 6500 cGy	6340.2	5684.2
TMjoints	D2% # < 7500 cGy	5352.6	5210.4
OralCavity	Dmean < 4500 cGy	3513.2	3369.3
ETbones	Dmean < 5200 cGy	5492.7	5316.7
IACs	Dmean < 4500 cGy	6574	6471.1
Larynx_Supraglottic	Dmean < 4500 cGy	3706.4	3561.7
Larynx_Glottic	Dmean < 4500 cGy	3792.9	3631.9
TympanicCavities	Dmean < 3400 cGy	4433.7	4702
VestibulSemis	Dmean < 4500 cGy	4045.6	3611.6

**Figure 3:**
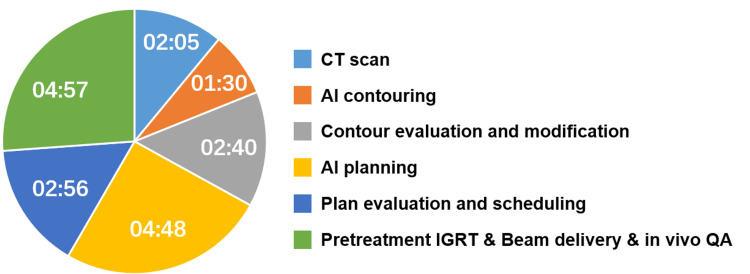
Duration of online adaptive workflow, with values presented as time in minute:second CT: Computed tomography; AI: Artificial intelligence; IGRT: Image-guided radiation therapy; QA: Quality assurance

Figure 3 displays the timing data for each step of the online ART process. The total treatment time measured from the FBCT scan to the end of treatment delivery was 18 min 56 s. AI contouring took 1 min 30 s, manual evaluation and modification of AI-generated contours took 2 min 40 s, and AI planning took 4 min 48 s. As we continue implementing online ART for more patients with NPC, we will collect data and provide a thorough statistical analysis in future studies.

The patient completed all treatments and achieved a complete response 12 weeks post-treatment according to the Response Evaluation Criteria In Solid Tumors version 1.1, with EBV DNA levels reduced to 0 copies/ml. This patient is currently alive without evidence of high-grade toxicity or local recurrence at approximately 10 months post-treatment.

## Discussion

In this case report, we described the first reported use of FBCT-guided online ART in a patient with NPC. Results indicate that online ART efficiently adjusts the radiotherapy plan. Furthermore, online ART optimized target volume coverage, particularly for PGTVn and PCTV2, and reduced the doses to most OARs. This case report provides evidence supporting the feasibility and dosimetric advantages of FBCT-guided online ART for NPC.

IMRT improves tumor control and reduces long-term radiation-induced complications, contingent upon accurate contouring and precise delivery of treatment plans [22]. A considerable proportion of patients with NPC still experience troublesome acute and late side effects. In grades 2 to 4 xerostomia and sensorineural hearing loss occur with 39.3% and 37.0% incidence, respectively [23]. One of the major reasons for the high rates of toxicity is the excessive dose of OARs during radiotherapy, as inter-fractional variations compromise treatment plans. While offline ART addresses some anatomical variations, it does not account for stochastic variations, necessitating online ART [24-27].

For head and neck cancers, the proximity of the target volumes to numerous critical organs poses significant challenges in treatment planning. Online ART requires the rapid design of high-quality plans while the patient is positioned on the treatment table, making it a complex and demanding task. Yoon et al achieved a median online ART plan generation time of 19.5 min in a retrospective simulated environment [17], while Vladimir et al. reported a mean online ART time of 26.1 min in a clinical environment for head and neck cancer [15]. Both studies excluded the time for patient setup, image acquisition, and treatment delivery. In a randomized trial, the average time radiation therapists spent at the console (from radiation therapist arrival to quality assurance approval.) was 22 min per online ART fraction [28]. For this patient, excluding CT scan and treatment delivery time, the total time for online ART was 11 min 54 s, confirming the feasibility of online ART using this novel integrated platform, and the efficiency and accuracy of AI contouring and AI planning.

The dosimetric superiority of online ART was observed in this patient. Online ART significantly improved PTV coverage and reduced the dose of most OARs, aligning with the dosimetric benefit seen in offline ART studies [10, 24 ,29]. In this case, the targets PGTVn and PCTV2 showed greater dose benefits than PGTVp and PCTV1. PGTVn and PCTV2, located in the cervical region, are more susceptible to setup errors and anatomical changes, which can compromise dose distribution. Thus, online ART offers more advantages for PGTVn and PCTV2. Whether the dosimetric superiority of online ART translates to clinical benefits, such as improved disease outcomes and quality of life or decreased side effects, remains to be seen. Retrospective data have reported that offline ART improved loco-regional recurrence-free survival and quality of life [24-27]. Mid-course or two-thirds course evaluation and offline replanning may offer similar dosimetric benefits without affecting treatment machine throughput or increasing patient stress. However, we emphasize the distinct advantages of online ART, which enables rapid adjustments within minutes, reducing the risks posed by anatomical changes that may occur during the days needed for offline replanning. Furthermore, online ART allows for multiple real-time adjustments throughout the treatment, potentially enhancing precision across the entire course of radiotherapy. We believe online ART represents a significant advancement in radiotherapy, aligning with current practices and future trends.

This patient is currently alive without evidence of high-grade toxicity or local recurrence at approximately 10 months post-treatment. Further long-term follow-up of this patient and others treated with online ART is needed to confirm the clinical benefits of online ART.

## Conclusions

We reported treating patients with NPC using FBCT-guided online ART on a novel integrated platform. This case demonstrated that online ART can rapidly optimize target volume coverage while reducing doses to OARs. Future research on a larger population is necessary to confirm these results and the clinical benefit of online ART by assessing survival outcomes, toxicity, and quality of life data.

## Appendices

Details of the uRT-linac 506c

We used the newly designed CT-integrated linac, the uRT-linac 506c, introduced by United Imaging Healthcare (UIH) Co., Ltd. This system differs from conventional CT-on-rails systems, which typically feature a sliding CT gantry and dual rotation axes on the couch top. Instead, the uRT-linac 506c integrates a diagnostic-quality helical CT system compactly fixed behind the gantry of a C-arm linac. The helical kilovolt (kV) FBCT employed by this system offers substantial advantages over the commonly used kV CBCT for image-guided radiotherapy. It is nearly free from degrading of photon scattering, which results in shading artifacts and deterioration of overall uniformity, providing a slice-to-slice comparison of a patient’s anatomy with planning CT and direct access to online ART with accurate CT number accounting for inter-fractional anatomic changes.

The uRT-linac 506c operates with a 6-MV treatment beam that can be delivered in two modes: flattened with a flattening filter (FF) and unflattened flattening-filter-free (FFF). The maximum dose rates are 600 MU/min for the FF mode and 1400 MU/min for the FFF mode. For imaging, the system uses a 1.5-MV imaging beam in FFF mode with a dose rate of 40 MU/min. Clinical electron beam modality is not available with this machine. The treatment head had dual-layered collimating jaws and 60 multi-leaf collimator (MLC) pairs. The MLCs have a width of 0.5 cm at the isocenter within the inner 20 cm and a width of 1.0 cm in the outer 20 cm, enabling a maximum field size of 40 × 40 cm².
